# Effectiveness of vonoprazan-based regimens compared with proton pump inhibitor-based regimens as first-line *Helicobacter pylori* agents

**DOI:** 10.3389/fphar.2023.1216433

**Published:** 2023-07-19

**Authors:** Toshihiko Kakiuchi

**Affiliations:** ^1^ Department of Pediatrics, Faculty of Medicine, Saga University, Saga, Japan

**Keywords:** *Helicobacter pylori*, vonoprazan, proton pump inhibitor, child, adult

## Abstract

In this study, we compared the success rate of eradicating Helicobacter pylori (*H. pylori*) in adults and children using vonoprazan (VPZ)-based *H. pylori* regimens to that of proton pump inhibitors (PPIs). In Japan, the success rate of a VPZ-based regimen as first-line therapy was lower in children than in adults. Compared with adults, children around puberty have higher CYP2C19 and CYP3A4 enzymatic activity to metabolize PPIs and VPZ. Further, children generally have shorter intestinal transit times than adults and may absorb antibiotics to a lesser extent. When comparing success rates of pediatric and adult eradication therapy using VPZ, it is very important to maintain a higher intragastric pH with sufficient gastric acid suppression to maintain *H. pylori* in a replicating state and amoxicillin and clarithromycin in the intestinal tract for as long as possible by reducing diarrhea as a side effect. Based on the above, it is reasonable that VPZ, which can suppress stomach acids more strongly than PPI, is a more relevant *H. pylori* eradication therapy.

## 1 Introduction

Previous research suggested that vonoprazan (VPZ)-based *Helicobacter pylori* (*H. pylori*) regimens for eradication therapy had a superior eradication efficacy than frequently used proton pump inhibitor (PPI)-based regimens in adults (Murakami et al., 2016; Lyu et al., 2019; Sun et al., 2022). VPZ is a new, potent acid-inhibitory drug, which competitively inhibits potassium binding to the hydrogen-potassium ATPase in gastric parietal cells more efficiently than PPIs (Abdel-Aziz et al., 2021). Therefore, VPZ-based regimens facilitate a shorter eradication therapy and potentially provide significantly higher eradication rates of clarithromycin-resistant strains. To examine why VPZ-based *H. pylori* regimens for eradication therapy were more effective in eradicating *H. pylori* than PPI regimens, we compared its success rate for *H. pylori* eradication in adults and children.

## 2 *Helicobacter pylori* eradication success rate between adults and children

Table 1 shows that the first-line *H. pylori* eradication therapy’s success rate by VPZ, amoxicillin (AMPC) and clarithromycin (CAM) for 7 days in Japan, and divided it into adults and children. All these data were previously reported. Considering regional *H. pylori* genotype, I intentionally limited the reports from Japan and omitted the results using second-line *H. pylori* eradication therapy, VPZ dual therapy, and metronidazole. VPZ-based regimen as first-line therapy’s success rate in children was lower than those in adults.

**TABLE 1:** List of manuscripts on *Helicobacter pylori* eradication therapy success rate using vonoprazan as first-line agent in Japan.

	Authors year	Study design	Number	Regimen	Drug doses VPZ, AMPC, CAM twice for day	Duration (days)	CAM Resistance rate	Success rate
Adult	Murakami et al. (2016)	RCT	329	VPZ + AMPC + CAM	20, 750, 200 or 400 mg	7	38.3%	92.6% (ITT)
	Suzuki et al. (2016)	ROS	181	VPZ + AMPC + CAM	20, 750, 200 mg	7	N/A	91.2% (PP), 89.1% (ITT)
	Tsujimae et al. (2016)	POS	439	VPZ + AMPC + CAM	20, 750, 200 mg	7	N/A	86.3% (PP), 84.6% (ITT)
	Tanabe et al. (2017)	ROS	363	VPZ + AMPC + CAM	20, 750, 200 or 400 mg	7	23.5%	94.4% (PP), 87.2% (ITT)
	Takara et al. (2019)	POS	189	VPZ + AMPC + CAM	20, 750, 200 mg	7	N/A	91.0% (ITT)
	Ozaki et al. (2018)	POS	1,688	VPZ + AMPC + CAM	20, 750, 200 or 400 mg	7	N/A	90.8% (ITT)
	Suzuki et al. (2020)	RCT	167	VPZ + AMPC + CAM	20, 750, 200 mg	7	25.6%	90.2% (PP), 89.2% (ITT)
Child	Kusano et al. (2017)	POS	118	VPZ + AMPC + CAM	20, 750, 200 mg	7	N/A	85.7% (PP), 81.3% (ITT)
	Kakiuchi et al. (2019)	POS	501	VPZ + AMPC + CAM	20, 750, 200 mg	7	N/A	85.1% (PP)
	Kaji et al. (2020)	POS	144	VPZ + AMPC + CAM	20, 750, 200 mg	7	N/A	83.8% (PP), 82.6% (ITT)
	Gotoda et al. (2020)	POS	161	VPZ + AMPC + CAM	20, 750, 200 mg	7	N/A	84.0% (PP), 81.9% (ITT)
	Kakiuchi et al. (2022)	POS	83	VPZ + AMPC + CAM	20, 750, 200 mg	7	57.8%	81.9% (ITT)
	Kakiuchi et al. (2023)	POS	836	VPZ + AMPC + CAM	20, 750, 200 mg	7	N/A	84.0% (PP), 73.6% (ITT)

RCT, randomized controlled trial; ROS, retrospective observational study; POS, prospective observational study; VPZ, vonoprazan; AMPC, amoxicillin; N/A, not applicable; CAM, clarithromycin; ITT, intention-to-treat; PP, per protocol.

## 3 Discussion

There were three possible reasons why the eradication rate of adults using VPZ was higher than that of children. First, adolescents tend to have a higher rate of *H. pylori* CAM resistance. It has been reported that the *H. pylori* CAM resistance rate is high among young people in Japan (Okamura et al., 2014), which is believed to be a result of the frequent CAM prescription use for childhood respiratory tract infections in this generation (Tsuboi and Iinuma, 2021). Among CAM-resistant strains, VPZ has a significantly higher eradication success rate than PPI (Murakami et al., 2016; Okubo et al., 2020), suggesting that it is important to suppress acid and make AMPC, not CAM, more effective. In addition, sufficient acid suppression is crucial for effectiveness because, bacteria enter their replicative state and become susceptible to AMPC and CAM at high pH. Second, children around puberty have higher CYP2C19 and CYP3A4 enzyme activity to metabolize PPIs and VAP (Anderson and Lynn, 2009). As a result, children metabolize VAP more rapidly than adults and may be attenuated at a higher rate than adults. Third, it has been reported that the ionized agent absorption (many antibacterial agents) that are poorly lipophilic is affected by the small bowel transit time (Martinez and Amidon, 2002). Children generally have shorter intestinal transit times than adults and may absorb less antibiotics than adults. This may be supported by our report (Kakiuchi et al., 2021) that diarrhea during eradication therapy reduces *H. pylori* eradication success rate. The former two suggests that sufficient gastric acid suppression is important for *H. pylori* eradication. The last reason suggests the importance of sufficient antibiotic absorption for *H. pylori* eradication. Graham (Graham DY, 2023) demonstrated that *H. pylori* eradication was successfully achieved by high intragastric concentrations of antibiotics or an intragastric pH at which amoxicillin is active.

In contrast, the efficacy of a VPZ-based regimen in a Western adult population was not higher compared with that in an Asian population (Chey WD et al., 2022). VPZ is mainly metabolized by CYP3A4 enzyme. Given the lack of clinically significant differences in VPZ pharmacokinetics between Asian and non-Asian populations, the differences between studies conducted among Western adults and Japanese populations are most likely not due to race alone. Different treatment compliance rates may also have contributed to outcome differences. With respect to children, there is no data regarding the eradication of *H. pylori* using VPZ in Western countries; therefore, we could not compare its eradication success rate between populations of Asian, including Japan, and Western countries.

In conclusion, a comparison of pediatric and adult eradication therapy success rates using VPZ could reinforce the following conclusions: with regard to *H. pylori* eradication therapy, it is very important to maintain a higher intragastric pH with sufficient gastric acid suppression to keep *H. pylori* in a replicating state and to keep AMPC and CAM in the intestinal tract as long as possible by reducing diarrhea as a side effect. It is reasonable that VPZ, which is more potent in stomach acid suppression than PPI, should gain a dominant position in *H. pylori* eradication therapy.

## Data Availability

The original contributions presented in the study are included in the article/Supplementary Material, further inquiries can be directed to the corresponding author.
